# LungCARE: Encouraging Shared Decision-Making in Lung Cancer Screening—a Randomized Trial

**DOI:** 10.1007/s11606-023-08189-1

**Published:** 2023-08-31

**Authors:** Judith M. E. Walsh, Leah Karliner, Ashley Smith, Yan Leykin, Steven E. Gregorich, Jennifer Livaudais-Toman, Ana I. Velazquez, Margaret Lowenstein, Celia P. Kaplan

**Affiliations:** 1 Department of Medicine, Division of General Internal Medicine, University of California, San Francisco (UCSF), San Francisco, CA USA; 2 Multi-Ethnic Health Equity Research Center, UCSF, San Francisco, CA USA; 3 Helen Diller Family Comprehensive Cancer Center, UCSF, San Francisco, CA USA; 4 Department of Psychology, Palo Alto University, Palo Alto, CA USA; 5 Department of Medicine, Division of Hematology/Oncology, UCSF, San Francisco, CA USA; 6 Division of General Internal Medicine, University of Pennsylvania Perelman School of Medicine, Philadelphia, PA USA; 7 Leonard Davis Institute of Health Economics, Philadelphia, PA USA

**Keywords:** lung cancer screening, shared decision-making

## Abstract

**Background:**

Lung cancer screening (LCS) is recommended for individuals at high risk due to age and smoking history after a shared decision-making conversation. However, little is known about best strategies for incorporating shared decision-making, especially in a busy primary care setting.

**Objective:**

To develop a novel tool, Lung Cancer Assessment of Risk and Education (LungCARE) to guide LCS decisions among eligible primary care patients.

**Design:**

Pilot cluster randomized controlled trial of LungCARE versus usual care.

**Participants:**

Patients of providers in a university primary care clinic, who met criteria for LCS.

**Intervention:**

Providers were randomized to LungCARE intervention or control. LungCARE participants completed a computer tablet-based video assessment of lung cancer educational needs in the waiting room prior to a primary care visit. Patient and provider both received a summary handout of patient concerns and responses.

**Main Measures:**

All eligible patients completed baseline interviews by telephone. One week after the index visit, participants completed a follow-up telephone survey that assessed patient-physician discussion of LCS, referral to and scheduling of LCS, as well as LCS knowledge and acceptability of LungCARE. Two months after index visit, we reviewed patients’ electronic health records (EHRs) for evidence of a shared decision-making conversation and referral to and receipt of LCS.

**Key Results:**

A total of 66 participants completed baseline and follow-up visits (34: LungCARE; 32: usual care). Mean age was 65.9 (± 6.0). Based on EHR review, compared to usual care, LungCARE participants were more likely to have discussed LCS with their physicians (56% vs 25%; *p* = 0.04) and to be referred to LCS (44% vs 13%; *p* < 0.02). Intervention participants were also more likely to complete LCS (32% vs 13%; *p* < 0.01) and had higher knowledge scores (mean score 6.5 (± 1.7) vs 5.5 (± 1.4; *p* < 0.01).

**Conclusions:**

LungCARE increased discussion, referral, and completion of LCS and improved LCS knowledge.

**Clinical Trial Registration:**

NCT03862001.

**Supplementary Information:**

The online version contains supplementary material available at 10.1007/s11606-023-08189-1.

## INTRODUCTION

Lung cancer is the leading cause of cancer death in the U.S. Survival rates increase dramatically with early diagnosis at an asymptomatic stage,^1^ highlighting the importance of early detection.^2^ Considerable effort has gone into identifying an accurate screening test.^3^ In 2013, based on the National Lung Screening Trial (NLST) results,^4^ the U.S. Preventive Services Task Force (USPSTF) recommended annual screening using low-dose computed tomography (LDCT) for high-risk individuals,^5^ updating those recommendations in 2021.^6^ Although screening reduces mortality, the high rate of false positives leads to additional testing and procedures.

Additionally, screening involves radiation exposure and, depending on comorbidities, may not be appropriate for all potentially eligible patients. Because of this, a shared decision-making approach to lung cancer screening (LCS) is recommended. In 2015, the Centers for Medicare and Medicaid Services (CMS) added annual screening with LDCT as a preventive service under Medicare for individuals at high risk, stipulating that the order for screening should occur following a shared decision-making visit.^7^

Although both the USPSTF and CMS stress the importance of shared decision about LDCT screening, primary care providers (PCPs), who are central to this, have limited tools to guide these discussions.^8,9^ Previously reported barriers include lack of appropriate patient education materials, lack of time, and complexity of decision-making.^10–12^ Existing informational content is highly variable and may be difficult to understand.^13^ Patients have challenges understanding individual risks and benefits of screening,^14,15^ and physicians may have difficulties determining patient eligibility and appropriateness of screening.^15,16^ Hence, prior studies have shown that shared decision-making conversations rarely happen^10,12^ resulting in a lack of LCS being offered to eligible patients. To address this implementation challenge, there is a critical need for strategies to facilitate shared decision-making discussions about LCS in primary care.

Prior studies have evaluated the efficacy of shared decision-making tools or patient decision aids on LCS outcomes. These include studies of individuals enrolled in specialty LCS programs,^17^ smokers accessing quit lines,^18^ or direct outreach to individuals potentially eligible for screening.^19^ However, these studies have not been conducted within primary care practices, where cancer screening is typically addressed. Previous studies in primary care settings have not compared the use of a shared decision-making tool with usual care. ^20,21^ Because primary care practices and providers are already handling many competing priorities aside from LCS, ^22^ it is critical to design tools that can be used in the real world to support primary care providers in addressing patients’ informational needs efficiently.

Based on our prior research exploring attitudes and priorities of patients and physicians in discussing LCS, we identified factors important in shared decision-making.^10^ With the input of patients and physicians, we developed Lung Cancer Assessment of Risk and Education (LungCARE), a patient-facing tool to promote discussion of lung cancer risk and facilitate shared decision-making about LCS in the primary care setting. Our aim was to evaluate the acceptability of LungCARE and its impact on the shared decision-making conversation and LCS outcomes among high-risk primary care patients and their PCPs.

## METHODS

### Design Overview

We conducted a pilot cluster randomized controlled trial of the LungCARE educational intervention versus usual care among patients eligible for LCS being seen in primary care. We assessed the impact of LungCARE on discussion of LCS, referrals and completion of screening, patient knowledge about LCS, and acceptability of the tool. All study procedures were approved by the UCSF Institutional Review Board.

### Study Setting

Our study took place in an academic general internal medicine practice in San Francisco, CA, serving approximately 26,000 diverse adults with a mix of public and private insurance. EPIC is the electronic health record used in this setting. As part of standard of care, all providers had access to a lung cancer screening “smart phrase.” Smart phrases allow commonly used chunks of text to be easily inserted into patient notes. The LCS smart phrase includes the key points to be addressed during a shared decision-making conversation.

### Participants

All PCPs (faculty, fellow and resident physicians, nurse practitioners) in the practice were invited to participate, and all agreed. Patients of these PCPs were eligible to participate if they met criteria for LCS and had a PCP visit scheduled during the study period of March 2019–March 2020 (age 55–80, spoke English, at least 30 pack-year smoking history, and were either current smokers or had quit within the past 15 years), and had a primary care appointment within the upcoming 2 weeks. Additional criteria included no prior history of lung cancer, no LDCT within the last year, ability to complete a telephone interview, and preferred language of English.

### LungCARE Intervention

LungCARE was designed to guide decision-making regarding LCS. We based the contents of LungCARE on the results of our qualitative study of attitudes and priorities of patients and physicians in real-world practice.^10^

LungCARE was administered on a touch tablet. Content of the intervention included the following: (1) what is lung cancer, (2) LCS overview, (3) how does screening work, (4) who is it for, (5) benefits of screening (early detection, early treatment), and (6) risks of screening (annual commitment, radiation exposure, false alarms). The programming was completed using Qualtrics XM software.^23^ LungCARE began with a 5-min animated video describing LCS, likelihood of incidental findings, risk of false positives, and pros and cons of LCS, as well as treatment and decision resources. After watching the video, patients completed risk and preference assessments, which included inclination toward screening, willingness to get LDCT, and their degree of agreement or disagreement with potential screening related concerns including : need for more information, willingness to accept a false positive result, concerns about getting a biopsy or additional tests, concerns about radiation, and the need for yearly repeat screening. All were encouraged to discuss screening with their providers.

After completion of the LungCARE risk and preference assessments, the patient was provided with two printed reports: (1) an individualized patient report (optimized for graphic appeal) written in plain language, and (2) an individualized report to hand to their physician (optimized for rapid scanning by clinicians) designed to efficiently prompt patient-physician discussion of LCS and including a link to the electronic health record (EHR) to simplify LCS screening referrals (see Appendices). These reports summarized willingness to undergo LCS and patients’ concerns with LCS. It also provided referral for smoking cessation resources, as appropriate.

### Randomization

Participating providers were randomized to LungCARE or usual care. We randomized at the physician/provider level to reduce intra-physician/cross-patient contamination. Using “restricted randomization,”^24^ we created two sets of participating providers matched on their characteristics: type (faculty, fellow, resident, nurse practitioner) and gender, and profiles of their panels (racial/ethnic and age distribution and rates of current and former smoking). One provider set was then randomly assigned to the LungCARE intervention (*n* = 22) and the other to usual care (*n* = 23).

### Recruitment

Eligible patients were identified using electronic health records (EHR), with research staff reviewing participating PCP schedules weekly to identify potentially eligible patients based on documented age and smoking history. We asked PCPs to review the list of potentially eligible patients and identify any patients not appropriate for recruitment (e.g., cognitive or psychiatric reasons). Recruitment letters, including an opt-out postcard, were mailed to all potentially eligible patients. One week after mailing, a LungCARE staff member telephoned those who did not opt-out; all potentially eligible patients were contacted. After confirming smoking history eligibility, patients were assigned to LungCARE intervention or usual care, based on group assignment of their PCP, and completed a baseline telephone survey.

Participants met with a LungCARE staff member 20 min prior to their scheduled primary care appointment. All signed a HIPAA release form authorizing the study team to review their EHR. Usual care patients continued to their visit while intervention patients received the LungCARE intervention, which they completed on a tablet in a quiet location. All participants were contacted within a week of their visit to complete the follow-up telephone survey. EHRs were reviewed 2 months after the index visit for evidence of a shared decision-making conversation, and the referral to and completion of LCS.

### Measures

#### Discussion and Screening Outcomes

The primary outcome was discussion of LCS as documented in the EHR. Secondary outcomes included referral for screening and completion of LDCT, as well as self-reported LCS knowledge on the follow-up survey.

EHR Outcome Measures:

- *Primary*: Two months after the index visit, we conducted a review of the patient’s EHR to identify documentation of screening discussion (yes/no).
- *Secondary*: EHR review also identified documentation of referrals for screening (LDCT referral made yes/no) and receipt of screening (LDCT completed yes/no).

Self-reported Outcome Measures:

- *Primary*: Patient-physician discussion of lung cancer risk and screening (discussed yes/no) on follow-up survey at 1 week post-index visit.
- *Secondary*: During the follow-up survey, patients were asked whether they were referred for LDCT (yes/no), and whether they had scheduled LDCT (yes/no). Patients were also asked a series of ten knowledge questions (true or false) about lung cancer. Topics included frequency of screening, likelihood of finding cancer or non-cancerous abnormalities, risks of radiation exposure, and screening eligibility. A summary knowledge score was created.

Combined Self-reported/EHR Outcome Measures:

We combined data from the follow-up survey and the EHR review to create outcomes describing LCS discussion, referrals, and LCS scheduled/completed.

- *Primary*: If patients answered “yes” to the question of whether they had discussed lung cancer risk and screening, or if there was a documentation of the discussion in the EHR, they were considered to have discussed LCS.
- *Secondary*: If patients answered “yes” to the question of whether they were referred to LCS at their appointment or if there was EHR documentation of an order for LCS, they were considered to have been referred for LCS. Similarly, if patients answered “yes” to whether they had scheduled LCS or if there was EHR documentation that they had completed LCS, they were considered to have scheduled/completed screening.

#### Intervention Acceptability

Among LungCARE intervention patients, we assessed acceptability of the intervention at two points. We asked two questions on the tablet with Likert scales immediately after watching the informational video: (1) how much they liked the video (1 “a lot,” 2 “a little,” 3 “not very much,” 4 “not at all,” 5 “I didn’t pay attention to the video”) and (2) how easy or difficult it was to understand the video (1 “very easy,” 2 “somewhat easy,” 3 “somewhat difficult,” 4 “very difficult,” 5 “I didn’t pay attention to the video”). At the follow-up phone call 1 week after the index visit, we asked participants about the individualized handout and report: (1) whether they remembered the handout and report (yes/no), (2) how they liked the handout and report, and (3) how easy or difficult it was to understand the information in the handout and report (see response categories above).

#### Smoking History, Family History, and Demographics

At baseline, all patients were asked about family history of lung cancer, prior LCS, and cigarette smoking history. The following demographic data were captured in the baseline survey: education, marital status, employment status, race/ethnicity, country of origin. Demographic data captured via EHR review was specific to 5 racial/ethnic categories (White/Caucasian, Black/African American, Hispanic/Latino/a, Asian, other).

### Statistical Analysis

Data analysis included descriptive statistics, using *t*-tests, and chi-square statistics to compare demographic characteristics between those randomized to intervention vs. usual care. Using intent-to-treat analyses, we compared LungCARE and usual care with respect to discussion of LCS screening, referral to and completion of screening, as well as associations between LCS knowledge and intervention status. Analyses accounted for clustering of patients within physicians. Analyses were conducted using Stata v.16.1.^25^

## RESULTS

### Recruitment

**Figure 1:**
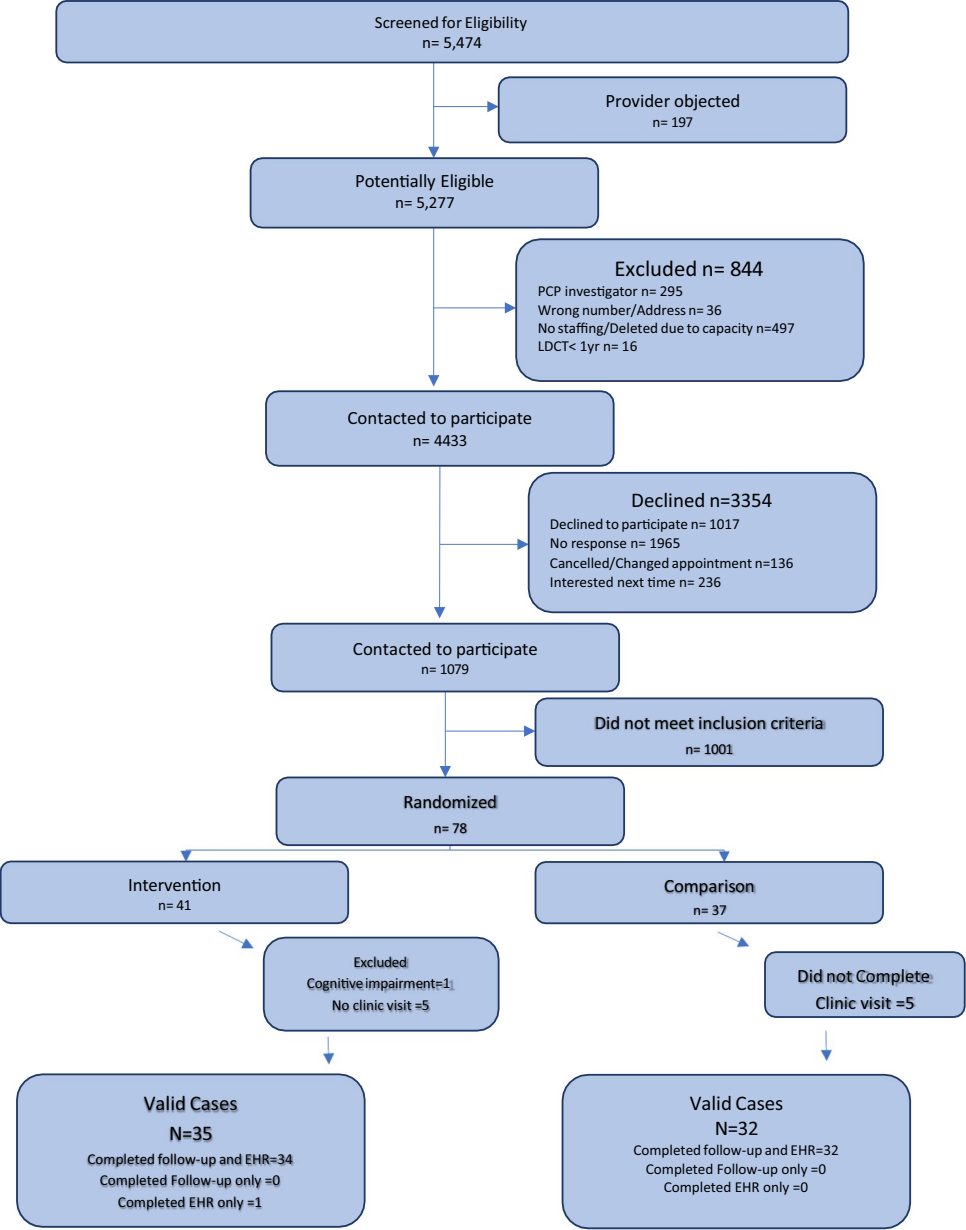
**CONSORT** **diagram.**

As shown in Fig. 1, we identified 5474 participants who met preliminary eligibility criteria. After exclusions, non-responses, and refusals, we confirmed smoking eligibility for 1079 potential participants; 1001 did not meet inclusion criteria for current or former smoking: 78 were enrolled and randomized per PCP group (LungCARE: *n* = 41; usual care: *n* = 37). Our goal was to recruit 120 participants. However, recruitment time was reduced by 2 months due to the COVID 19 pandemic. Of 78 patients randomized, 66 (85%) completed baseline and follow up visits (Fig. 1).

### Description of Study Population

At baseline, intervention and usual care groups were well-balanced with respect to demographic and health characteristics (Table 1). Mean age was 65.9 (SD = 6.0) years, 62% were White, 30% were African American, and 5% were Asian. The LungCARE group reported more education and was less likely to report a family history of lung cancer.

**Table 1:** Baseline Demographic Characteristics of LungCare participants by Randomization Group (among Participants who completed baseline and follow-up surveys) (*N* = 66)

	Control (*N* = 32) *N* (%)*	Intervention (*N* = 34) *N* (%)*	Total (*N* = 66) *N* (%)*	*p*-value
Age				
(Mean ± SD)	64.9 ± 5.7	66.7 ± 6.3	65.9 ± 6.0	0.27
Gender				
Female	18 (56)	16 (47)	34 (52)	0.49
Male	14 (44)	17 (50)	31 (47)	
Non-binary	0	1 (3)	1 (2)	
Race/ethnicity				
White/Caucasian	19 (59)	22 (65)	41 (62)	0.92
Black/African American	10 (31)	10 (29)	20 (30)	
Asian	2 (6)	1 (3)	3 (5)	
Other	1 (3)	1 (3)	2 (3)	
Country of birth				
US born	31 (97)	29 (85)	60 (91)	0.11
Foreign born	1 (3)	5 (15)	6 (9)	
Education				
Less than high school	4 (13)	2 (6)	6 (9)	**0.02**
High school or equivalent	5 (16)	5 (15)	10 (15)	
Some college	18 (56)	10 (29)	28 (42)	
College or beyond	5 (16)	17 (50)	22 (33)	
Health literacy				
Inadequate	6 (19)	7 (21)	13 (20.0)	0.79
Adequate	26 (81)	26 (79)	52 (80.0)	
Marital status				
Married/living with long-				
term partner	7 (21.9)	10 (29.4)	17 (25.8)	0.50
Other	25 (78.1)	24 (70.6)	49 (74.2)	
Smoking status				
Current	9 (28)	9 (27)	18 (27)	0.87
Former	23 (72)	25 (74)	48 (73)	
Family history of lung				
cancer (parents,				
siblings, children)				
No	20 (63)	30 (88)	50 (76)	**0.01**
Yes	12 (38)	4 (12)	16 (24)	

^*^Percentages based on non-missing values. Some do not add to 100% due to rounding

### LCS Discussion

Using data from the EHR, participants in LungCARE were more likely to have discussed LCS with their physicians (56% vs 25%: *p* = 0.04). Combining data from self-report and EHR review, LungCARE participants were more likely to have discussed LCS with their PCP, although this was not statistically significant (62% versus 38%: *p* = 0.08) (Table 2).

**Table 2:** Lung cancer screening discussion, referral, and completion of LCS among LungCare participants

	Control *N* = 32 *N* (%)	Intervention *N* = 34 *N* (%)	*p*-value
Lung cancer screening **discussed** during visit (**EHR review**)	8 (25)	19 (56)	**0.04**
Lung cancer screening **discussed** during visit (**self-report or EHR review**)	12 (38)	21 (62)	0.08
Doctor **referred** patient for lung cancer screening (**EHR review**)	4 (13)	15 (44)	**0.02**
Doctor **referred** patient for lung cancer screening (**self-report or EHR review**)	6 (19)	18 (53)	** < 0.01**
Patient **completed** lung cancer screening (**EHR review**)	4 (13)	11 (32)	**0.01**
Patient **scheduled/completed** lung cancer screening (**self-report or EHR review**)	5 (16)	11 (32)	0.08

### LDCT Referral, and Scheduled/Completed LDCT

Using data from the EHR, participants in LungCARE were more likely to be referred for LDCT (44% vs 13%: *p* = 0.02). In addition, they were more likely to schedule or complete LDCT (32% vs 13%: *p* = 0.01). Combining data from self-report and EHR review, participants in LungCARE were more likely to be referred to LDCT (53% vs 19%, *p* < 0.01), and to schedule or complete LDCT, although the latter difference was not statistically significant (32% vs 16%, *p* = 0.08) (Table 2).

### LCS Knowledge

LungCARE participants had higher overall knowledge scores at follow-up than those in usual care (mean score 6.5 versus 5.5, *p* < 0.01). LungCARE participants were more likely to answer correctly that both false positive and false negative screening tests could occur (Table 3).

**Table 3:** Knowledge about lung cancer and screening after the intervention among LungCare Participants

	*N* (%) correct answers		
Knowledge of lung cancer and screening (Correct answer in parentheses)	Control *N* = 32	Intervention *N* = 34	*p*-value
Lung cancer screening should be done every 2 years (FALSE)	7 (22)	15 (44)	**0.04**
Lung cancer screening can miss cancer in the lungs.(TRUE)	13 (41)	26 (77)	** < 0.01**
All abnormalities found in the lungs turn out to be cancer (FALSE)	25 (78)	30 (88)	0.25
Without screening, lung cancer is usually found when it is more advanced and less likely to be cured. (TRUE)	27 (84)	25 (76)	0.35
“False-alarm” scans may show that you have a cancer when you actually do not. (TRUE)	15 (47)	29 (85)	** < 0.01**
All smokers should be screened for lung cancer. (FALSE)	1 (3)	5 (15)	0.11
Lung cancer screening can cure cancer. (FALSE)	27 (84)	29 (85)	0.92
Radiation exposure is one of the harms of lung cancer screening. (TRUE)	18 (56)	20 (59)	0.83
Lung cancer screening lowers the chances of developing lung cancer. (TRUE)	17 (53)	10 (29)	**0.03**
Former smokers do not need to be screened for lung cancer. (FALSE)	27 (84)	32 (94)	0.22
Knowledge score: Total number of correct answers out of 10 (mean ± SD)	5.5 ± 1.4 [range: 3–8]	6.5 ± 1.7 [range: 3–9]	** < 0.01**

### Acceptability of LungCare

The majority (78%) of intervention participants liked the video “a lot” and nearly all (91%) reported that it was “very easy” to understand. Over half of participants (59%) liked the personalized patient handout and report “a lot” and 78% reported that the handout and report were “very easy” to understand.

## CONCLUSIONS

We evaluated the acceptability and impact of a primary care focused intervention (LungCARE) to promote provider-patient discussion and LCS shared decision-making among eligible patients. LungCARE resulted in higher likelihood of provider-patient discussions of screening, referrals for screening, and completion of LCS. LungCARE also resulted in greater patient knowledge, suggesting that the information provided was sufficient (despite the tool’s concise format), and participants gave the components of the LungCARE tool positive ratings.

Our study adds to the literature in several ways. First, LungCARE was effective in increasing discussions about, referrals for and completion of LCS, when compared with usual care. Despite strong evidence for efficacy of LCS in reducing mortality,^4^ there are substantial implementation gaps, with only 4–7% of eligible US adults screened.^26,27^ The need for improvements in implementation has been heightened in light of recent USPSTF recommendations expanding screening eligibility by lowering the eligibility age from 55 to 50 and the pack-year smoking history from 30 to 20.^6^ Communication challenges, including need for conversations about individual risks and benefits and shared decision-making, have been cited as key barriers to implementation.^10,12,28^ PCPs have cited a lack of patient educational materials or communication tools as an additional barrier.^10,12^ LungCARE increased patient knowledge about LCS. This is consistent with other shared decision-making tools that addressed knowledge.^17,18,21^ However, unlike those studies, LungCARE also demonstrated increases in screening referrals relative to usual care. This suggests that implementing LungCARE in the primary care setting may be an effective model for facilitating shared decision-making and assisting patients to make choices more aligned with evidence.

Other important aspects of LungCARE were its implementation involving three levels of the health care structure (patient, provider, and system) and its effectiveness in the primary care setting.^29^ Other studies have evaluated patients accessing quit lines^18^ or in mixed primary care and specialty settings^17^ and either did not increase or did not evaluate rates of testing. One reason for the success of LungCARE may be its incorporation into a primary care visit, including making changes in the EHR, which included a smart phrase accessible to all clinicians which addressed components of the LCS shared decision-making conversation, to make decisions actionable for patients and providers. Primary care is a critical venue for screening discussion since it may be the first or only contact that an individual has with the health care system. Further, interventions targeting smokers via quit lines may miss high-risk, eligible former smokers who may not access smoking cessation resources but do receive primary care services. While primary care is an important setting for screening discussions, it can also be a challenging one because there are many competing priorities including acute and chronic illnesses and other health care maintenance tasks. ^22^ A shared decision-making conversation takes time, especially when done within the context of a visit where many other issues are being addressed. Part of the success of LungCARE may come from being administered just before the visit, highlighting this issue among competing priorities and priming patients for the discussion with their PCP.

Patients expressed largely positive impressions of the key components of LungCARE, namely the video and personalized handout and report. The almost uniform acceptability of the short video points to the easy format and accessible language. However, brief survey measures may not fully capture the patient experience; additional studies should explore patient experiences in depth via interviews and observations of how the tool is used in practice. Future work should also explore acceptability and impact of LungCARE from a provider perspective to fully optimize the use of LungCARE tool in the primary care setting.

Given the complexities of understanding personal lung cancer risk,^30,31^ screening risks and benefits, ^6^ and recommendations for screening follow-up,^6^ a concise and acceptable format for a decision tool targeting patients is critical. Comparable results were found in a study of a similarly delivered intervention addressing breast cancer risk reduction.^32,33^

This study has limitations. This was a single-site study, and recruitment was cut short in March of 2020, at the beginning of the COVID-19 pandemic, when all in-person clinical research was halted. As a result, we were unable to reach our recruitment target. Despite this, we identified significant differences in key outcomes.

Further, although the implementation of LungCARE in the primary care clinic was feasible, it required the screening of a large number of patients due to strict eligibility criteria for LDCT and limited patient smoking data at the clinical practice.

Despite these challenges, we recruited a sufficient number of participants to conduct a pilot cluster randomized trial. Overall, the intervention was well accepted and improved key indicators including screening discussion, referral to and completion of screening, and knowledge of LCS. If reproducible, the increase in rates of referral to and completion of screening seen in this trial could have a significant impact on larger scale lung cancer screening effectiveness. Future directions include refinement of the tool, particularly in the context of new USPSTF recommendations, which expand the number of individuals eligible for screening^6^, as well as testing the intervention in a larger trial across multiple primary care settings.

### Supplementary Information

Below is the link to the electronic supplementary material.

Supplementary file1 (PDF 494 kb)

## Data Availability

The authors confirm that the data supporting the findings of this study are available within the article [and/or] its supplementary materials.
