# Supplementary material for: LungCARE: Encouraging Shared Decision-Making in Lung Cancer Screening—a Randomized Trial
Source: J Gen Intern Med. 2023 Aug 31;38(14):3115–22. doi: 10.1007/s11606-023-08189-1 (PMC10651593; doi:10.1007/s11606-023-08189-1)
Supplement: Supplementary file 1 — Supplementary file1 (PDF 494 kb) [file 11606_2023_8189_MOESM1_ESM.pdf]

Appendix

1. Sample Patient Report
2. Sample Clinician Report

We thank you for your time spent taking this survey.

Your response has been recorded.

Below is a summary of your responses Download PDF

PLEASE HAND THE iPAD BACK

TO THE RESEARCHER

PERSONALIZED REPORT FOR LUNG CANCER SCREENING

Date: Wednesday, February

20th Dear Jan,

Summary of Responses:

Lung Cancer Screening Eligibility

Based on your age and smoking history, you are eligible for lung cancer screening.

Screening preference

You indicated that you...

- would like to get screened for lung cancer.

- You are thinking about quitting smoking within the next 6 months.
  - Talk to your doctor to learn more about how s/he can help you quit.
  - Ask your doctor about the UCSF Fontana Tobacco Treatment Center and get additional information from The California Smokers Helpline toll free at 1-

Other responses

You are concerned about.

- radiation from lung cancer screening.
- having lung cancer screening done every year for at least 3 years.

You are not concerned about.

Patient Preferences for Lung Cancer Screening

A DECISION AID FOR LUNG CANCER SCREENING

-
-

PATIENT INFORMATION

Jan

Wednesday, February 20th

ELIGIBILITY

[illegible]

FOR PATIENT DOCUMENTAION, GUIDELINES, COUNSELING, AND REFERRALS

-
- Lung Cancer Screening SmartPhrase: .gmclinlungcancerscreening

SCREENING PREFERENCE

The patient indicated that she...

-
-

RISK FACTORS

-
- current smoker
- smoked, on average, 20 cigarettes per day for 30 years.
- seriously considering quitting smoking within the next 6 months.
- at least one first-degree relative diagnosed with lung cancer

OTHER RESPONSES

The patient is concerned about...

- radiation exposure
- annual screens

The patient is not concerned about...

Please log in.

RecipientExternalDataReference	7005
